# Considering chronotype to improve hypertension management

**DOI:** 10.1016/j.eclinm.2024.102768

**Published:** 2024-07-26

**Authors:** Tomas Baka, Darlynn M. Rojo-Wissar, Fedor Simko

**Affiliations:** a Institute of Pathophysiology, Faculty of Medicine, Comenius University, Bratislava, Slovak Republic; b Department of Psychiatry and Human Behavior, Warren Alpert Medical School of Brown University, Providence, RI, USA; c Bradley/Hasbro Children's Research Center, E.P. Bradley Hospital, Providence, RI, USA; d 3^rd^ Department of Internal Medicine, Faculty of Medicine, Comenius University, Bratislava, Slovak Republic; e Institute of Experimental Endocrinology, Biomedical Research Center, Slovak Academy of Sciences, Bratislava, Slovak Republic

Hypertension is the dominant risk factor for cardiovascular pathologies, yet it remains poorly controlled. Chronotherapy, an approach considering circadian (i.e., ∼24-h) rhythms in physiology and pathology, could contribute additional benefit to hypertension management.

Chronotherapy of hypertension considers the circadian variations in blood pressure (BP).^1^ People with attenuated systolic BP decline during the night (non-dippers) or night-time relative systolic BP increase are recognised to be at elevated cardiovascular risk.^2^ Multiple prospective trials indicate that adverse cardiovascular events are better predicted by asleep systolic BP mean than by the daytime or 24-h ambulatory BP means.^3^ The Hygia Chronotherapy Trial encompassing 19,084 hypertensive patients showed that ingesting the entire daily dose of antihypertensive medications at bedtime, as opposed to upon awaking, was associated with a significant reduction of cardiovascular events and increased survival time. This benefit was brought about by decreasing the asleep systolic BP mean and improving the sleep-time relative systolic BP decline towards the dipper ambulatory BP pattern.^4^

Efficacy of the chronotherapeutic approach need not only be associated with the therapeutic modification of hemodynamic alterations, but the concomitant interference with neurohumoral activation, peripheral organ remodelling, insulin sensitivity, platelet aggregability or lipid metabolism, which may be influenced by individual circadian variations of gene expression.^5^ Thus, it should be taken into account that the effect of pharmacological modification of BP and related pathologies may also be dependent on one’s chronotype. Chronotype reflects the timing of an individual’s circadian rhythms, which is determined by inherent endogenous molecular clocks as well as environmental and societal cues. Indeed, people with morningness chronotype tend to get up early and are more active in the morning, whilst people with eveningness chronotype tend to get up later and are more active in the evening. The available data suggest that the eveningness chronotype is associated with elevated cardiometabolic risk rendering chronotype a prospective, yet overlooked, cardiovascular risk factor.^6^

Recently in eClinicalMedcine, Filippo Pigazzani and colleagues report their findings from the Chronotype sub-study of the Treatment in Morning versus Evening (TIME) study, which investigated the combined effect of timing of antihypertensive drugs and chronotype on cardiovascular outcomes in patients with arterial hypertension.^7^ Chronotype was assessed quantitatively by the modified Munich ChronoType Questionnaire and qualitatively by self-reporting. In line with previous literature, eveningness chronotype was associated with increased incidence of non-fatal myocardial infarction (MI) or stroke. The results showed lower incidence of non-fatal MI when the antihypertensive medication dosing time aligned with the patient’s chronotype, i.e. eveningness chronotype dosed in the evening and morningness chronotype dosed in the morning. Strikingly, dosing time misalignment with chronotype was associated with increased risk for non-fatal MI. However, no effect of hypertension chronotherapy on the risk of non-fatal stroke was observed, potentially due to low non-fatal stroke incidence in the sub-study. In patients with intermediate chronotype, representing about 50% of the sub-study population, no effect of dosing time on cardiovascular events was shown. This observation corresponds with the results of the original TIME study showing no difference between evening vs morning dosing of antihypertensive drugs with respect to vascular death and non-fatal MI or stroke.^8^ Overall, these results suggest that chronotype assessment could improve hypertension risk stratification and management and reduce the risk of adverse cardiovascular events by better tailoring the dosing time of antihypertensive drugs.

The Chronotype sub-study which respects the patient’s chronotype in terms of timing the antihypertensive medication, could refine the therapeutic approach to hypertension. However, one should take into consideration the potential pitfalls in conducting chronotherapeutic studies^9^ when interpreting these findings. Interesting results of the Chronotype sub-study are warranted confirmation in larger prospective clinical trials with more diverse populations and elevated cardiovascular risk to broaden generalisability and deepen the mechanistic understanding of the chronotype—hypertension therapeutic implications. Furthermore, a more rigorously controlled selection of patients and consideration of biological time instead of arbitrary hourly time intervals could strengthen the value of the results. Additionally, use of ambulatory BP monitoring in future studies may help detect isolated office hypertension or masked hypertension and control for hemodynamic effects of therapeutic intervention.

In addition to BP, elevated heart rate and its non-dipping are also significant cardiovascular risk factors that are generally neglected in hypertensive patients.^10^ Therefore, it seems reasonable to contemplate that concurrent consideration of chronotype and circadian heart rate variations could better cardiovascular risk assessment and heart rate control in hypertension, and thus potentially improve cardiovascular prognosis.

The Chronotype sub-study provides proof-of-concept evidence that considering endogenous circadian rhythms by estimation of the hypertensive patient’s chronotype could help finetune cardiovascular risk stratification and antihypertensive therapy tailoring to reduce the risk of adverse cardiovascular events.

## Contributors

TB and FS: conceptualisation, literature search, writing-original draft, review and editing. DMR: literature search, writing-review and editing. All authors approved the submitted version.

## Declaration of interests

DMR will receive an honoraria for coauthoring a report for the Abell Foundation on school start times in Baltimore, MD. She is currently a DEI Committee member for the Sleep Research Society, and was the Trainee Member at Large on the board of directors from 2022 to 2023. FS received honoraria for presentations from Novo Nordisk Slovakia, Egis Slovakia and Roche Slovakia. TB has no conflicts of interests to declare.
